# Immunization and Cardiovascular Disease in Latin America. The CorVacc Study: Sub Analysis by Regions

**DOI:** 10.5334/gh.1600

**Published:** 2026-11-12

**Authors:** Fernando Wyss, Daniel Piskorz, Ricardo Lopez-Santi, Lucelli Yañez Gutiérrez, Horacio Márquez-Gonzales, Fabio Enrique Parada, Vladimir Ullauri, Ana Munera Echeverri, Juan Esteban Gómez, Osiris Valdez, Máxima Méndez, Claudia Almonte, Carlos Ponte-Negretti, María Alayde Mendoça Rivera, Iván Romero Rivera, Raúl Villar, Jorge Alberto Rivera, Dulman Pineda de Paz, Ana Isabel Barrientos, Edmundo Jordan, Gonzalo Piskorz, Pilar Lopez-Santi, On Behalf of CorVacc study investigators. Cardiovascular Prevention Council (SIAC-PREVENT) of the Inter-American Society of Cardiology (SIAC)

**Affiliations:** 1 Cardiovascular Services and Technology of Guatemala, Guatemala; 2 Instituto de Cardiología del Sanatorio Británico, Rosario, Argentina; 3 Hospital Italiano de la Plata, La Plata, Argentina; 4 Congenital Heart Disease Department, National Medical Center 21st. Century, México; 5 Department of Cardiology, Instituto Guatemalteco de Seguridad Social (IGSS), Guatemala, Guatemala; 6 Hospital Metropolitano de Quito, Universidad Internacional del Ecuador, Quito, Ecuador; 7 Clínica Rosario Tesoro – Cardioestudio, Medellín, Colombia; 8 Fundación Valle de Lili, Unidad de Cardiología, Cali, Colombia; 9 Hospital Central de la Romana, La Romana, Republica Dominicana; 10 Cli-Lipid, Santo Domingo, República Dominicana; 11 CEMDOE Unidad de Cardiología, Santo Domingo, República Dominicana; 12 Instituto Medico la Floresta, Caracas, Venezuela; 13 Hospital Universitario de la Universidad Federal de Alagoas, Maceió, Alagoas, Brazil; 14 Universidad Federal de Alagoas, Alagoas, Brazil; 15 GINERAD Salud, La Serena, Chile; 16 CARDIOMEDIC, Guatemala, Guatemala; 17 CARDIOCLÍNIC, Unidad de Cardiología, Hospital Centro Médico Zacapa, Guatemala, Guatemala; 18 Hospital Bendaña, San Pedro Sula, Honduras; 19 Hospital Pavía Santurce Cardiology Center, San Juan, Puerto Rico; 20 Data & Analytics, Data IQ, Buenos Aires, Argentina; 21 Leiden University Medical Center, Leids Universitair Medish Centrum, Leiden, Holanda, The Netherlands

**Keywords:** Vaccines, Influenza, Pneumococcus, Regions, North, Central America, Caribbean, Andean, Southern Cone, Cardiovascular Disease

## Abstract

**Introduction:**

In Latin America, the mortality rate from influenza and pneumococcal disease has increased in recent years. Several challenges limit the implementation of vaccination strategies. These factors contribute to lower-than-expected immunization rates in Latin America.

**Objective:**

The objective of this sub-analysis of the Inter-American Registry of Influenza and Pneumococcal Vaccination (CorVacc) is to determine vaccination rates and analyze the factors that influence the North – Central America and Carribean Spanish Speaking (NCC); Andean region (AR) and South Cone (SCR).

**Methods:**

A cross-sectional survey of the general population was conducted in 19 Latin American countries. Adults aged 18 and older completed a 34-question online survey. Data were grouped into seven categories: demographic, socioeconomic, and non-demographic. Multivariate logistic regression comparisons were performed between regions to assess the association between vaccination rates and their explanatory variables.

**Results:**

Influenza, Pneumococcus and ≥3 doses of COVID-19 vaccination was received by 4,126 (45.18%) CAC, 3,576 (50, 13%) AR and 3,024 (62, 99%) SC; 5,904 (65, 09%) CAC, 5,162 (68, 9%) AR and 3,486 (72, 61%) SC; and 6,266 (68, 61%) CAC, 6,001 (80, 09%) AR and 2,892 (79, 99%), respectively. No association was detected between the regions and the likelihood of receiving the influenza vaccine: AR OR 0.94 (95% CI 0.84–1, 06; p = 0.33), CAC OR 0.81 (CI 0.80–1, 00; p = 0.053). On the contrary, a positive association was detected with Streptococcus pneumoniae vaccination: AR OR 1.12 (CI 1, 01–1, 24; p = 0.032), CAC OR 1.33 (CI 1, 20–1, 47; p < 0.001). A negative association was observed in CAC with COVID-19 ≥ 3 doses vaccination: OR 0.56 (CI 0, 65; p = 0.032; p < 0.001). Social, economic and confidence barriers were identified, but cardiovascular risk factors and comorbidities were not significantly associated with lower vaccination rates.

**Conclusions:**

Social, economic, and cultural, but not cardiovascular risk factor or disease variables in Latin American region could explain the lower-than-expected rates of vaccination and the differences observed between regions in vaccination frequencies.

## Graphical Abstract

**Figure:**
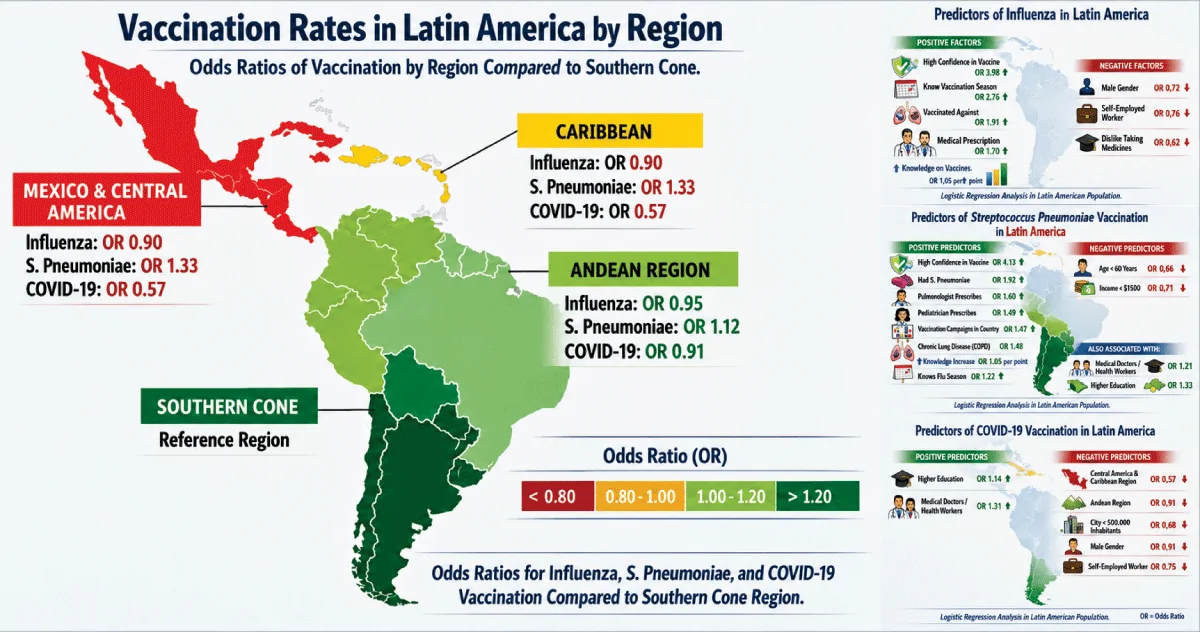


## Introduction

Cardiovascular (CV) disease is the leading cause of morbidity and mortality worldwide. Current global health policy goals include achieving a 25% reduction in premature mortality from non-communicable diseases (NCDs) by 2025 (1).

There is a strong association between respiratory infections and acute CV events. All strains of influenza and Streptococcus pneumoniae infections can trigger a range of CV alterations that may lead to hospitalization or death (2,3,4,5,6). Evidence has shown that influenza vaccination (IV) and pneumococcal vaccination (PV) are associated with reduced rates of several CV outcomes, including myocardial infarction (MI), heart failure (HF) hospitalization, and CV mortality (7,8,9,10,11,12,13,14,15,16,17).

Despite these benefits, several challenges limit the implementation of vaccination strategies in Latin America. Barriers include patient-related factors (vaccine hesitancy, prior experiences, misinformation), healthcare providers’ knowledge and attitudes toward vaccination, and healthcare system constraints. Collectively, these factors contribute to lower-than-expected immunization rates in the region (18,19,20,21,22,23,24,25,26,27).

In 2023, region of the Americas made commendable efforts to reinvigorate vaccination as a public good, and most of them have succeeded in increasing coverage. However, there are still opportunities for improvement, with a view to achieving the vaccination coverage levels recorded at the beginning of this century and meeting the region’s established targets (28). Vaccination trend in Latin America among health workers, pregnant women, and those with chronic diseases are lower than expected in the whole region (29).

There is evidence of low vaccination coverage and a high incidence rate of pneumonia in older adults, due to insufficient planning of prevention activities by the health facility, insufficient dissemination campaigns to the target population, and limited access to health services for the vulnerable population, which predisposes to low awareness and vaccination of this vulnerable age group; resulting in a directly proportional increase in cases of pneumonia with increasing age. It is estimated that pneumococcal bacteria cause between 12,000 and 28,000 deaths, 182,000 hospitalizations, and 1.4 million medical consultations each year in Latin America and the Caribbean. Countries in this region are among the first developing countries to have introduced pneumococcal conjugate vaccines (29).

Public health strategies based on an understanding of the epidemiological realities of each region and country in Latin America are essential to effectively address the barriers that impede the implementation of cardiovascular prevention projects based on vaccination. The CorVacc Study provides, for the first time, comprehensive and detailed information on the barriers and challenges that Latin American health systems face regarding vaccination (30).

The objective of this sub-analysis of the Inter-American Registry of Influenza and Pneumococcal Vaccination (CorVacc) was to identify regional rates differences and the factors influencing these rates.

## Methods and Design

### Study population

CorVacc was an observational study. A total of 19 countries prospectively enrolled in the Inter-American Registry of Influenza and Pneumococcal Vaccination (CorVacc Study). Outpatients aged 18 years or older provided informed consent to participate in the survey. Patients under 18 years of age and those unable to provide informed consent were excluded from the study (31).

### Informed consent

Patients were informed about the purpose of the survey and the anonymity of their responses. No personally identifiable information was collected. Ethical approval was obtained from the Research Ethics Committee of the Inter-American Society of Cardiology (SIAC) (31).

### Study design

A cross-sectional online survey comprising 34 questions was developed using Google Forms (Mountain View, CA). The research team invited patients to complete the questionnaire in person, by email, on social media, by telephone, or by paper questionnaires. The survey was divided into three sections:

1. Questions that examine a patient’s demographic profile.
2. Questions that examine their CV risk profile.
3. Questions that examine their vaccination profile.

The questionnaire consisted of a variety of question types, including dichotomous, Likert-type, multiple-choice, and open-ended response choices. The respondents had to answer all the questions, and they could select multiple responses, in accordance with the content of the question (31).

### Survey

A survey platform was created for patient enrollment. To improve the response rate, periodic reminders were sent by mail, text message, or social media. In addition, information on the evolution of the surveys by country was made available. The survey consists of 34 questions (Appendix), which has been published in the Mexican Archives of Cardiology, Immunization and Cardiovascular Disease in Latin America. The CorVacc study: rationale and design (30).

The validity and reliability of the survey were assessed using Cronbach’s coefficient and exploratory factor analysis.

### Operational variables

The Influenza vaccination was considered appropriate when it was held annually and in season, while the criteria for Streptococcus pneumoniae vaccine when two doses pneumococcus vaccine were received in the last 4 years, and COVID-19 vaccine when the subject received at least three doses.

### Study organization

Cardiologists and other physicians from Latin American countries were invited to join a team of collaborators for the CorVacc Study team. A total of 19 countries were represented by cardiologists who registered to participate in the study. The countries will be divided according to a prespecified geographic distribution as follows (Figure 1):

- North, Central, and Caribbean region (NCC): Mexico, Guatemala, El Salvador, Honduras, Nicaragua, Costa Rica, Panamá, Cuba, Puerto Rico, and Dominican Republic.
- Andean region (AR): Venezuela, Colombia, Ecuador, Bolivia, and Perú.
- Southern Cone region (SCR): Paraguay, Chile, Uruguay, Brazil, and Argentina.

**Figure 1:**
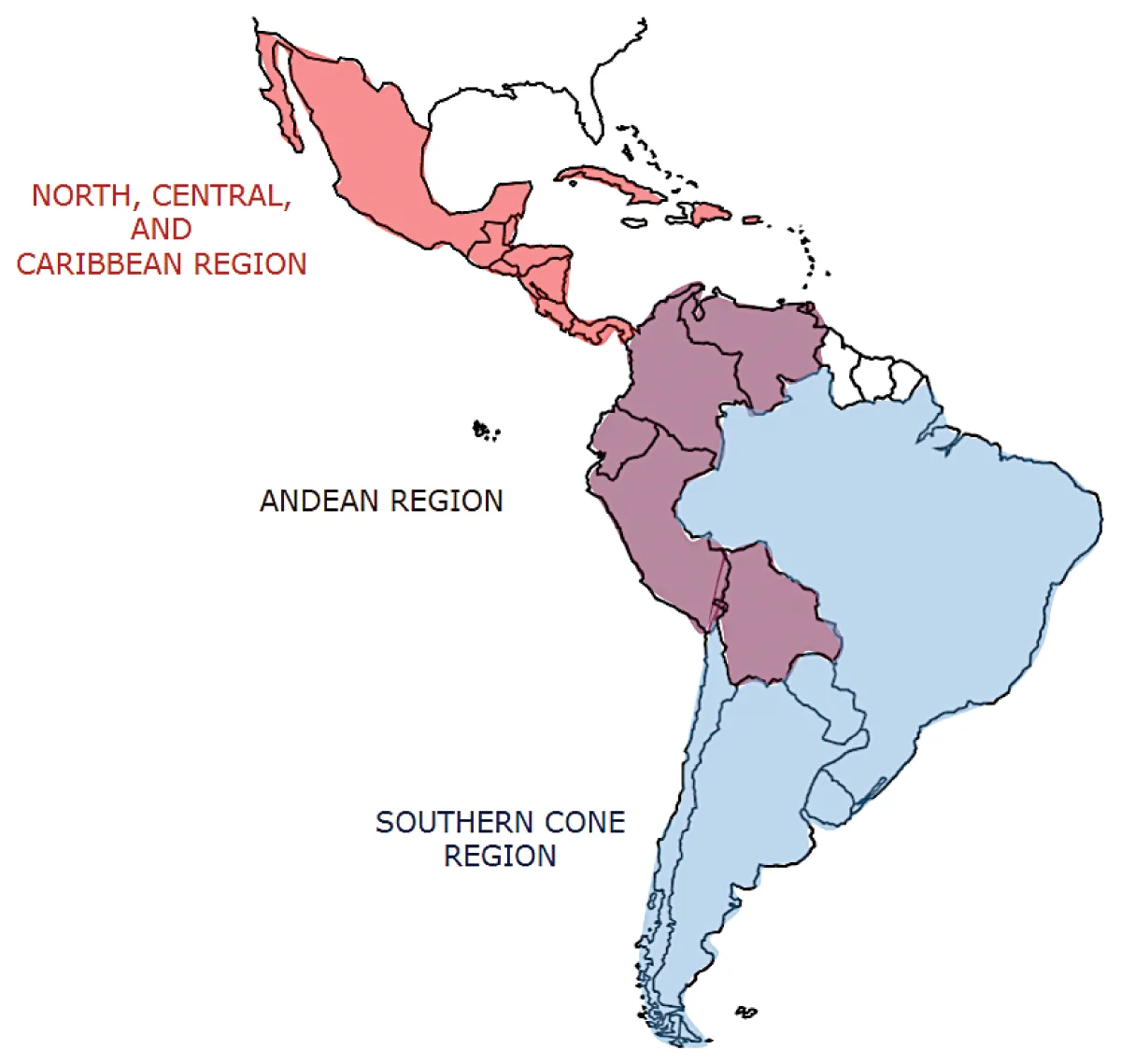
Distribution of participating countries by region (30).

### Statistical analysis

Continuous variables were presented as mean (standard deviation) if normally distributed, and as median (interquartile range) if they did not follow a normal distribution. Normality of distribution was assessed with Shapiro-Wilk and Kolmogorov-Smirnov tests. Categorical variables were expressed as percentages and compared between individuals with and without vaccination using the Pearson χ^2^ test. Vaccine confidence and knowledge were categorized with a Likert-scale from 0 to 10, 0 being no confident/knowledge at all and 10 being highly confident/knowledge. Multivariate logistic regression comparisons were performed between regions to assess the association between confidence and knowledge in vaccines and their explanatory variables. Appropriate vaccination against Influenza, Streptococcus pneumoniae, and COVID-19 was considered the dependent variable, while all others were considered independent variables. Goodness-of-fit and discrimination is reported as McFadden’s pseudo-R². The AUC is informed with a 95% CI and a p-value for the test against AUC = 0.50, using the Hanley-McNeil approximation for the standard error. The Likert-scale is made up of discrete variables, so the sample was separated into two categories according to whether the responses were ≥7, considered as general confidence/knowledge level, an expression of moderate-high standard, and ≥9, a more restrictive cut-off value, an indicator of full confidence/knowledge. All statistical analyses were performed using SPSS for Windows, version 29.0 (IBM Armonk, NY, USA), and a two-tailed p-value <0.05 was considered statistically significant.

The total population of Latin American countries was estimated at approximately 662 million. A sample size of 20,000 surveys was calculated to achieve a margin of error of 5%, a confidence level of 90%, and an error of distribution of 50%.

## Results

The CorVACC-AMERICA study included 21,425 subjects, 8,093 from Central America and Caribbean Spanish speaking, 7,492 from Andean region, and 4,801 from South Cone. Significant regional differences were identified in demographic profile, cardiovascular risk factors, access to healthcare system, therapeutic burden and knowledge about cardiovascular vaccination benefits between the three regions (Table 1).

**Table 1:** Baseline characteristics of the sample.


VARIABLE	CENTRAL AMERICA & CARIBBEAN	ANDEAN REGION	SOUTHERN CONE	p VALUE

N	8,093	7,492	4,801	

Influenza vaccination (n – %)	3,304–40.8%	3,756–50.1%	2,284–47.57%	< 0.001

Streptococcus pneumoniae (n-%)	2,727–33.7%	2,611–34.85%	1,498–31.2%	0.05

≥ 3 doses COVID-19 (n-%)	6,161–76.13%	5,948–79.39%	3,832–79.82%	< 0.001

Female gender (n-%)	5,382–66.5%	4,647–62.03%	3,033–63.17%	< 0.001

Mean age (age + STD)	45.29 + 17.48	45.81 + 16.17	46.51 + 16.42	0.025

Locality of residence				

100,000–500,000 inhabitants	1,459–18%	951–12.69%	1,201–25.02%	< 0.001

> 500,000 inhabitants	2,408–29.75%	3,272–43.69%	1,866–38.87%	< 0.001

Employment relationship				

Active	6,352–78.49%	5,749–70.09%	2,963–61.72%	< 0.001

Retired	1,204–14.88%	877–11.71%	647–13.48%	0.41

Health service				

Public	2,175–26.87%	2,197–21.18%	1,847–31.37%	< 0.001

Social Security	3,750–46.3%	3,290–31.72%	805–13.67%	< 0.001

Cardiovascular risk factors				

Hypertension	2,858–35.3%	1,603–21.4%	1,252–26.08%	< 0.001

Overweight/Obesity	1,681–20.8%	1,457–19.45%	1,020–21.25%	0.07

Dyslipidemia	1,143–14.1%	1,098–14.66%	778–16.2%	NS

Diabetes	1,082–13.4%	451–6.02%	573–11.94%	NS

Smoking	273–3.37%	240–3.2%	231–5.02%	< 0.001

Comorbidities				

Coronary artery disease	505–6.24%	328–4.38%	143–2.97%	< 0.001

Heart failure	296–3.66%	250–3.34%	78–1.62%	< 0.001

Kidney disease	172–2.13%	159–2.12%	73–1.52%	< 0.001

Stroke	98–1.21%	113–1.51%	39–0.81%	< 0.001

Medication use				

None	4,155–45.5%	4,001–53.4%	2,040–42.49%	< 0.001

≥ 3 medications	2,049–25.48%	1,257–16.77%	1,074–22.38%	< 0.001

Knowledge about cardiovascular benefits of vaccination	1,851–22.87%	1,793–29.48%	971–31.91%	< 0.001


The multivariate logistic regression analysis showed that no statistically significant differences in influenza vaccination frequencies were detected across the three regions: Andean region OR 0.945 (95% CI 0.842–1.06; p = 0, 335), Central America and Caribbean region OR 0.895 (95% CI 0.80–1.001; p = 0.053). Higher education and postgraduate individuals, male gender and self-employed workers showed a lower likelihood of being vaccinated. On the other side, confidence on Influenza vaccine ≥7 and those subjects that know vaccination season have the higher likelihood. A very interesting point is that when the vaccine is indicated by a cardiologist the probability of vaccination increases to about 90%. In this same direction, type 2 diabetes patients have a higher likelihood of almost 30%. The area under the curve (AUC) of the model was 0.87 (95% CI 0.865–0.875), R^2^ McFadden 0.350, p < 0.001 when the confidence on Influenza vaccination was ≥7 and 0.869 (95% CI 0.864–0.873), R^2^ McFadden 0.349, p < 0.001when the confidence was ≥9 (Table 2).

**Table 2:** Multivariate logistic regression for influenza vaccination.


VARIABLE/CATEGORY	β COEFFICIENT	95% CI β COEFFICIENT	OR	95% CI	p VALUE

Andean region	–0.057	–0.172 to 0.058	0.945	0.842–1.06	0.335

Central America & Caribbean region	–0.111	–0.223 to 0.001	0.895	0.800–1.001	0.053

Medical doctors and health and health care workers	0.306	0.204 to 0.409	1.358	1.226–1.505	< 0.001

Male gender	–0.330	–0.411 to –0.249	0.719	0.663–0.780	< 0.001

Self-employed worker	–0.280	–0.386 to –0.174	0.756	0.679–0.841	< 0.001

Mean financial income < US $1,500	0.168	0.044 to 0.292	1.183	1.045–1.399	0.008

I do not like to take medicines	–0.480	–0.794 to –0.167	0.619	0.452–0.847	0.003

Yes, I have Influenza	0.099	0.013 to 0.186	1.104	1.013–1.204	0.025

Confidence on Influenza vaccine ≥7	1.382	1.258 to 1.507	3.985	3.517–4.515	< 0.001

Yes, I have been vaccinated against Streptoccocus Pneumoniae	0.644	0.567 to 0.722	1.905	1.763–2.059	< 0.001

Family doctor prescribes vaccination	0.216	0.047 to 0.385	1.241	1.048–1.469	0.012

Cardiologist prescribes vaccination	0.530	0.295 to 0.764	1.698	1.343–2.148	< 0.001

1-point increase knowledge benefits vaccination	0.048	0.036 to 0.060	1.049	1.037–1.061	< 0.001

Yes, I know Influenza vaccination season	1.013	0.928 to 1.099	2.755	2.528–3.002	< 0.001

Yes, there are Influenza and Streptococcus pneumoniae vaccination campaigns in my country	0.522	0.380 to 0.663	1.685	1.463–1.941	< 0.001

Type 2 Diabetes	0.256	0.118 to 0.395	1.292	1.125–1.484	< 0.001


The multivariate logistic regression analysis showed that both regions, Andean OR 1.121 (95% CI 1.01–1.244; p = 0, 032) and Central America and Caribbean OR 1.331 (95% CI 1.201–1.475; p < 0.001) have higher likelihood of receiving Streptococcus pneumoniae vaccine than the South Cone. Age ≥60 years and those with a financial income < US $1,500 showed a lower likelihood of being vaccinated. On the contrary, confidence on Streptococcus pneumoniae vaccine ≥7 and those subjects with history of pneumococcus infection have the higher likelihood. If the vaccine is indicated by a pneumonologist or the pediatrician, the probability of vaccination increases to about 50 to 60%. In this same direction, chronic obstructive pulmonary disease patients have a higher likelihood of almost 48%. The AUC of the model was 0.782 (95% CI 0.776–0.789), R^2^ McFadden 0.194, p < 0.001 when the confidence on Influenza vaccination was ≥7 and 0.783 (95% CI 0.777–0.790), R^2^ McFadden 0.194, p < 0.001 when confidence was ≥9 (Table 3).

**Table 3:** Multivariate logistic regression for pneumococcal vaccination.


VARIABLE/CATEGORY	β COEFFICIENT	95% CI β COEFFICIENT	OR	95% CI	p VALUE

Andean region	0.114	0.010 to 0.216	1.121	1.010–1.244	0.032

Central America & Caribbean region	0.286	0.183 to 0.389	1.331	1,201–1.475	< 0.001

Higher education and postgraduate studies	0.132	0.034 to 0.230	1.141	1.034–1.259	0.009

Medical doctors and health care workers	0.192	0.098 to 0.285	1.211	1.103–1.330	< 0.001

Age ≤ 60 years	–0.412	–0.623 to –0.200	0.663	0.536–0.819	< 0.001

Mean financial income < US $1,500	–0.341	–0.455 to –0.228	0.711	0.635–0.796	< 0.001

Yes, I have Streptococcus pneumoniae	0.654	0.487 to 0.821	1.924	1.628–2.273	< 0.001

Confidence on Streptococcus pneumoniae vaccine ≥7	1.419	1.292 to 1.545	4.131	3.641–4.688	< 0.001

Pulmonologist prescribes vaccination	0.472	0.211 to 0.733	1.603	1.234–2.082	< 0.001

Pediatrician prescribes vaccination	0.399	0.141 to 0.657	1.490	1.151–1.929	0.002

1-point increase knowledge benefits vaccination	0.045	0.034 to 0.055	1.046	1.035–1.057	< 0.001

Yes, I know Influenza vaccination season	0.202	0.114 to 0.289	1.223	1.121–1.335	< 0.001

Yes, there are Influenza and Streptococcus pneumoniae vaccination campaigns in my country	0.386	0.250 to 0.523	1.472	1.264–1.687	< 0.001

Chronic obstructive pulmonary disease	0.389	0.196 to 0.581	1.475	1.217–1.788	< 0.001


The multivariate logistic regression analysis showed that Central America and Caribbean region (OR 0.568 (95% CI 0.493–0, 653; p < 0.001) has a significantly lower likelihood of receiving at least three doses of COVID-19 vaccine. Living in a city with <500,000 inhabitants, male gender and self-employed workers showed a lower likelihood of being vaccinated. The AUC of the model was 0.850 (95% CI 0.844–0.856), R^2^ McFadden 0.273, p < 0.001 when the confidence on Influenza vaccination was ≥7 and 0.846 (95% CI 0.841–0.852), R^2^ McFadden 0.258, p < 0.001when it was ≥9 (Table 4).

**Table 4:** Multivariate logistic regression for COVID-19 vaccination.


VARIABLE/CATEGORY	β COEFFICIENT	95% CI β COEFFICIENT	OR	95% CI	p VALUE

Andean region	–0.093	–0.245 to 0.058	0.911	0.783–1.059	0.225

Central America & Caribbean region	–0.566	–0.707 to –0.426	0.568	0.493–0.653	<0.001

Higher education and postgraduate studies	0.127	0.013 to 0.241	1.135	1.013–1.272	0.029

Medical doctors and health care workers	0.273	0.143 to 0.403	1.314	1.154–1.496	<0.001

Living in city <500.000 inhabitants	–0.387	–0.559 to –0.214	0.679	0.572–0.807	<0.001

Male gender	–0.100	–0.196 to –0.004	0.905	0.822–0.996	0.041

Self-employed worker	–0.286	–0.443 to 0.129	0.751	0.642–0.879	<0.001


## Discussion

Statistically significant differences were identified between Latin America regions in Streptococcus pneumoniae and COVID-19 vaccination rate. The likelihood of receiving Streptococcus pneumoniae vaccine is 12% and 33% higher in Andean and Central America and Caribbean regions, respectively, than in the South Cone. In addition, the probability of receiving at least three doses of the COVID-19 vaccine is 43% lower in Central America and Caribbean region.

Male gender and self-employed workers have a lower likelihood of receiving Influenza vaccine. Independent workers in Latin America lack health insurance coverage; therefore, vaccination will depend on provision by the public health system or their ability to pay out-of-pocket. Contrary, subjects that know Influenza vaccination season and the availability of Influenza and Streptococcus vaccination campaigns in their countries have a significantly higher probability of receiving Influenza vaccine. This information could be relevant to assist the health authorities of Latin American countries to adequately focus vaccination campaigns. In this same scenario, a moderate-high standard Influenza vaccine confidence and knowledge level was the most relevant variable associated to almost four times increase likelihood of being vaccinated. Prescription by a family physician or cardiologist is another variable associated with a higher probability of receiving the Influenza vaccine. These latest data underscore the role of healthcare professionals in promoting vaccination strategies and highlighting their individual and population-level benefits. Between the comorbidities, just type 2 diabetes is associated to higher Influenza vaccination.

A variable that could explain the low vaccination rate against Streptococcus pneumoniae in Latin America is low income. Individuals with an income lower than US $1,500 have a significantly 29% lower probability of being vaccinated against this bacterium. This finding, as in the case of influenza vaccination, reaffirms the need for social and economic support in the region. Again, the role played by the healthcare professional in recommending Streptococcus pneumoniae vaccination is essential, as the likelihood increases when it is recommended by a pulmonologist or pediatrician by 50 to 60%. Also, a moderate-high standard Influenza vaccine confidence and knowledge level was the most relevant variable associated to more than four times increase probability of being vaccinated. Patients with chronic obstructive pulmonary disease have a statistically significant 47% higher likelihood of being vaccinated against Streptococcus pneumoniae.

Living in small cities (with fewer than 500,000 inhabitants), being male, and being self-employed worker are associated with a lower probability of receiving the COVID-19 vaccine in Latin American regions.

The Inter-American Registry of Influenza and Pneumococcal Vaccination (CorVacc) showed clearly the huge challenges that the region faces in terms of cardiovascular prevention.

The risk of a heart attack increases sixfold in the week after a confirmed Influenza infection, and nearly one in eight people experienced an acute cardiovascular event. The risk of acquiring Influenza infection and its complications is more relevant in old and very old people, those with compromised immune systems, pregnant women, children under five years old and health system workers (32,33). Patients that received the Influenza vaccine had a 34% lower risk of major adverse cardiovascular events, and individuals with recent ACS had a 45% lower risk (34). Influenza vaccination is recommended with high level of evidence for individuals ≥65 age and for those who have experienced an acute coronary syndrome within the past year; a recommendation supported by a somewhat lower level of evidence applies to patients with chronic coronary syndromes or heart failure (35). In the CorVacc Registry some demographic, cultural and financial variables, but not regional aspects, were involved in the likelihood of being vaccinated. Previous registries have shown differences in the rate of vaccination in Latin America, while in absolute terms such evidence may be correct, the multivariable adjustment of this study shows data to the contrary (27). On the other hand, the region presents common barriers and opportunities for influenza vaccination. Community education campaigns could open a window of opportunity to improve vaccination rates, considering that vaccine confidence, knowledge of its benefits, and the existence of vaccination campaigns and the knowledge of the vaccination season have been positively associated with the likelihood of getting vaccinated in CorVacc Registry. No less important is the medical attitude, particularly the cardiologists, toward vaccination. In this study, when the vaccine was indicated by practitioners with this specialty the probability of receiving the vaccine was significantly higher.

Pneumococcal vaccine reduces the risk of pneumonia in adults aged ≥65 years and subjects with lung disease. The current recommendations for pneumococcal vaccination include adults aged ≥50 years who have not previously received the vaccine and all adults aged 19 to 49 years with a chronic medical risk condition (36,37). The CorVacc Registry shows results aligned with these recommendations as shown in Table 2, but Latin American cardiology specialists do not appear to be particularly involved in recommending the vaccine. This could explain why cardiovascular conditions such as coronary artery disease or heart failure did not show an association with the likelihood of being vaccine.

A meta-analysis that included 155 studies showed consistently that SARS-CoV-2 infection was associated with an increased risk of coronary heart disease (RR, 1.74 [95% CI, 1.44–2.11]) and stroke (RR, 1.69 [95% CI, 1.23–2.31]) (38). Randomized clinical trials showed that COVID-19 vaccine reduces the infection and its severity, while observational studies demonstrated a reduced incidence of myocardial infarction, stroke and atrial fibrillation (39,40). National and international guidelines recommend COVID-19 vaccination in patients who are moderately or severely immunocompromised to reduce mortality and in subjects with chronic coronary syndromes, and per public health to reduce infection complications (41). Medical doctors and health care workers are at 31% higher likelihood of receiving the vaccine in the CorVacc Registry, despite that, practitioner’s specialty is not associated to the indication to their patients.

Vaccine hesitancy and vaccine resistance challenge vaccination opportunities. Between the factors that could explain these attitudes lower socioeconomic status, societal norms, historical context and prior vaccination experience should be considered. Misinformation regarding vaccine safety and efficacy are between the main barriers. Education is the most effective method for mitigating vaccine hesitancy. Vaccines accessibility and affordability must be given serious consideration. Individuals are more likely to receive vaccination when it is recommended by a health care provider. In this context, cardiovascular specialists could play a key role in reducing missed opportunities (42). All these aspects were addressed by the Inter-American Registry of Influenza and Pneumococcal Vaccination (CorVacc).

### Limitations

This was a self-reported survey with convenience sampling, which raises concerns about potential bias with the sampling. Certain groups—such as individuals living in rural areas or small towns, or those with limited access to health services or digital tools—may be underrepresented. While these limitations must be considered, the nature of the multivariate analysis mitigates them to some extent. In this scenario, limitations regarding the generalizability of the observed data should be considered since the study population consists of outpatients participating voluntarily in the survey. Nevertheless, the sample size exceeded expectations and provides meaningful insights into Latin American respondents’ beliefs, expectations, and perceptions of vaccination.

## Conclusions

Vaccination rate, adherence to vaccination schedules, and confidence levels for Influenza, Streptococcus pneumococcus, and COVID-19 are critically lower than expected in Latin America. Significant differences between regions in the likelihood of Streptococcus pneumoniae were detected, it was more likely in Central America and Caribbean and Andean regions. On the contrary, COVID-19 vaccine was significantly less probable in Central America and Caribbean region than in the South Cone and Andean region. Work status, financial income and education level, could be considered common barriers in Latin America to accessibility and affordability. On the other side, vaccine confidence, knowledge of vaccination benefits, vaccination season and campaigns availability increase the probability of receiving this preventive tool. Practitioners, particularly cardiologists, play a key role recommending vaccination. These data could be of help for the regional design of public health and cardiovascular prevention strategies. All stakeholders, health funders and public health authorities, must take active steps to ensure availability, affordability, education, and adherence to schedules vaccination.

## Additional File

The additional file for this article can be found as follows:

10.5334/gh.1600.s1 Appendix. Description of the questions included in the survey.
