# Supplementary material for: Immunization and Cardiovascular Disease in Latin America. The CorVacc Study: Sub Analysis by Regions
Source: Glob Heart. 2026 Nov 12;21(1):81. doi: 10.5334/gh.1600 (PMC13761834; doi:10.5334/gh.1600)
Supplement: Supplementary file 1 [file gh-21-1-1600-s1.pdf]

Anexo: Description of the questions included in the survey.

Questions	Possible responses
Region	• North, Central America & the Caribbean<br>• Andean<br>• Southern Cone
Age	
Gender	• Female<br>• Male<br>• Transgender<br>• Other
What perception do you have of your state of health?	Scale of 1–10 (Unhealthy—Very healthy)
Number of inhabitants city/town of residence	• < 10,000 population<br>• 10,000–100,000 population<br>• 100,000–500,000 population<br>• > 500,000 population<br>• Don’t know
Marital relationship	• Married<br>• Divorced<br>• Single<br>• United to<br>• Widower<br>• None of the above

Work relationship	• Dependent assets<br>• Independent assets<br>• Retired/Pensioned/Retired<br>• Independent worker<br>• Irregular worker<br>• None of the above
Education level	• None<br>• Primary<br>• Secondary<br>• University<br>• Master's degree
If you are a professional, could you describe your occupation?	• Lawyer<br>• Business Administration<br>• Architect<br>• Engineer<br>• Entrepreneur<br>• Finance<br>• Doctor<br>• Marketing/Advertising<br>• Business<br>• Health area personnel<br>• Others
Economic income in US dollars per month	• < 499<br>• 500–999<br>• 1000–1499<br>• 1500–1999<br>• 2000–2499<br>• 2500–2999<br>• > 3000

Access to health services	• Private with direct payment<br>• Private with medical insurance<br>• Social Security<br>• Public Health Service<br>• None
What Chronic Diseases do you have?	• Cancer<br>• Mellitus diabetes<br>• Diseases of the Immune System<br>• Psychiatric mental illness<br>• Psychological<br>• Chronic lung diseases<br>• Renal disease<br>• Fatty liver<br>• Arterial hypertension<br>• Hypothyroidism<br>• Polycystic ovary<br>• Cholesterol and/or triglyceride problems<br>• Overweight/Obesity<br>• Smoking<br>• None of the above
Have you been diagnosed by a doctor with the following diseases?	• Angina pectoris<br>• Arrhythmias<br>• Disease of the arteries of the lower limbs and/or aorta<br>• Stroke<br>• Myocardial infarction<br>• Heart failure<br>• None of the above

Have you had any of the following procedures?	• Cardiac catheterization with stent placement in any coronary artery<br>• Cardiac surgery for heart valve prostheses<br>• Bypass surgery or revascularization of coronary arteries<br>• Lower limb vascular surgery<br>• Pacemaker<br>• None of the above
For his chronic illnesses, I take my medications.	• Every day without fail, at the corresponding time<br>• Every day without fail, but not at the corresponding time<br>• I remember from time to time<br>• I forget from time to time<br>• I don't take them<br>• I don't need medication
How many medications do you take?	• 1<br>• 2<br>• 3<br>• 4<br>• 5<br>• More than 5<br>• None<br>• I don't like taking medications
Please select your medication scheme	• Single-drug pills<br>• One pill with two medications<br>• One pill with three medications<br>• They told me it's called Polypill.<br>• None of the above

Have you had the flu?	• Yes<br>• No<br>• Don’t know
Have you had Pneumococcus?	• Yes<br>• No<br>• Don’t know
How confident are you in the Influenza vaccination?	Scale 1–10 (Not at all confident—Very confident)
How confident are you in the Pneumococcus vaccination?	Scale 1–10 (Not at all confident—Very confident)
How often do you get the Influenza vaccine?	• Annually in season<br>• Every 2 years<br>• Every 3 years<br>• When I remember<br>• From time to time<br>• I don’t put it on
Have you had a 2 doses pneumococcus vaccine in the last 4 years?	• Yes<br>• No
Who prescribes the vaccines?	• General Physician<br>• Internal Medicine<br>• Family Medicine<br>• Cardiologist<br>• Endocrinologist<br>• Geriatrician

	• Gynecologist<br>• Pulmonologist<br>• Neurologist<br>• Pediatrician for my children/grandchildren<br>• Others (Gastroenterologist, Rheumatologist, etc.)<br>• I prescribe myself<br>• None of the above
What health service does vaccination provide you?	• Public<br>• Private<br>• Social Security<br>• Annual vaccination campaign in my country<br>• I buy it at the pharmacy without prescription.<br>• None of the above
How much do you know about the cardiovascular benefits of vaccination?	Scale 1–10 (I don't know them—I know them)
If you are not a doctor, do you suggest other Vaccinations?	• Yes<br>• No<br>• Does not apply
If you are a doctor, do you indicate vaccination for Influenza and Pneumococcus?	• Yes<br>• No<br>• Does not apply
Do you know when the Influenza season is in your country?	• Yes<br>• No

Are there vaccination campaigns for Influenza and Pneumococcus in your country?	• Yes<br>• No<br>• Don't know
Have you been vaccinated for COVID-19?	• Yes<br>• No
Number of doses given.	• 1<br>• 2<br>• 3<br>• 4<br>• 5<br>• None<br>• All necessary
What type of vaccine do you use?	• Abdala (Cuban)<br>• Moderna<br>• J&J/Janssen<br>• Oxford/Astra Zeneca<br>• Pfizer/BioNTech<br>• Sinopharm (China)<br>• Sinovac/Biotech (China)<br>• Sputnik 5 (Russia)<br>• None
How confident are you in the COVID-19 vaccination?	Scale 1–10 (Not at all confident—Very confident)
